# Dietary Factors Modulate Iron Uptake in Caco-2 Cells from an Iron Ingot Used as a Home Fortificant to Prevent Iron Deficiency

**DOI:** 10.3390/nu9091005

**Published:** 2017-09-12

**Authors:** Ildefonso Rodriguez-Ramiro, Antonio Perfecto, Susan J. Fairweather-Tait

**Affiliations:** Norwich Medical School, University of East Anglia, Norwich NR4 7UQ, UK; a.perfecto@uea.ac.uk (A.P.); s.fairweather-tait@uea.ac.uk (S.J.F.-T.)

**Keywords:** iron bioavailability, iron fortification, simulated gastrointestinal digestion

## Abstract

Iron deficiency is a major public health concern and nutritional approaches are required to reduce its prevalence. The aim of this study was to examine the iron bioavailability of a novel home fortificant, the “Lucky Iron Fish™” (LIF) (www.luckyironfish.com/shop, Guelph, Canada) and the impact of dietary factors and a food matrix on iron uptake from LIF in Caco-2 cells. LIF released a substantial quantity of iron (about 1.2 mM) at pH 2 but this iron was only slightly soluble at pH 7 and not taken up by cells. The addition of ascorbic acid (AA) maintained the solubility of iron released from LIF (LIF-iron) at pH 7 and facilitated iron uptake by the cells in a concentration-dependent manner. In vitro digestion of LIF-iron in the presence of peas increased iron uptake 10-fold. However, the addition of tannic acid to the digestion reduced the cellular iron uptake 7.5-fold. Additionally, LIF-iron induced an overproduction of reactive oxygen species (ROS), similar to ferrous sulfate, but this effect was counteracted by the addition of AA. Overall, our data illustrate the major influence of dietary factors on iron solubility and bioavailability from LIF, and demonstrate that the addition of AA enhances iron uptake and reduces ROS in the intestinal lumen.

## 1. Introduction

The World Health Organization (WHO) estimated in 2010 that iron deficiency anemia (IDA) affects one third of the world’s population [1]. IDA is particularly prevalent in developing countries [2] and therefore represents a heavy economic burden. Amongst the strategies used to reduce the prevalence of iron deficiency, food-based or home fortification strategies can be very cost-effective [3].

Cooking in iron pots has been proposed as a strategy for improving the iron status of iron deficient populations [4]. However, its effectiveness is somewhat reduced by a lack of acceptability [5]. A recent study carried out in three refugee camps in Tanzania reported low acceptability for using iron and iron-alloy cooking pots due to a number of factors including rusting, heavy weight, difficulty in use and cleaning [6]. A new home fortification approach uses an iron ingot, the “Lucky Iron Fish™” (LIF), and has recently been tested in a Cambodian population [7,8,9]. It is based on the principle of releasing iron during cooking, as occurs with iron pots, but the LIF is much smaller, only weighing approximately 200 g, and has been shaped as a fish, a symbol of luck in Cambodian culture, in an attempt to improve its acceptability in this population [10]. Three randomised clinical trials (RCT) have been performed evaluating the effectiveness of LIF in reducing iron deficiency [7,8,11], with conflicting results. Apart from compliance issues related to its acceptability, other parameters, such as the composition of the diet or genotype, may have influenced the outcome of those trials. Therefore, there is a need to study the cellular iron bioavailability of this novel home fortificant and potential interactions with dietary factors.

The in vitro digestion/Caco-2 cell model has been extensively used to predict iron bioavailability from food and iron supplements and to investigate the intestinal cellular mechanisms of iron uptake [12,13,14,15]. Therefore, the aims of the present study were to use this model to evaluate the potential bioavailability of iron from LIF, taking into consideration the impact of dietary factors, and to examine oxidative stress initiated by the iron released from LIF, in order to provide new insights into this novel home iron fortificant.

## 2. Materials and Methods 

### 2.1. Samples and Reagents

**Figure 1:**
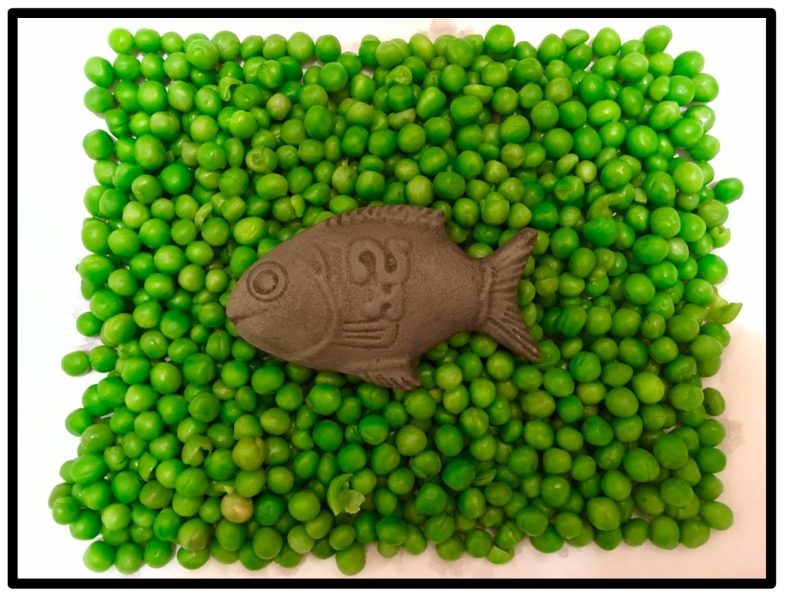
Iron ingot (Lucky Iron Fish™ (LIF)) used to treat iron deficiency. The selected picture background shows the relative size of LIF compared to *petit pois* peas.

The iron-ingot, Lucky Iron Fish™ (Figure 1), was purchased through an e-commerce online shop (www.luckyironfish.com/shop, Guelph, Canada). The same iron ingot was used for all experiments, cleaned in Milli-Q H_2_O, and dried at the end of each experiment. Chemicals, enzymes and hormones were purchased from Sigma-Aldrich, (Gillingham, UK) unless otherwise stated. Frozen *petit pois* peas (*Pisum sativum*) were obtained from a local supermarket, microwaved, lyophilized, finely ground and stored in a desiccator at 4 °C over silica gel. 

### 2.2. Cell Culture and LIF Treatments

Caco-2 cells (HTB-37) were obtained from the American Type Culture Collection (Manassas, VA, USA) at passage 20 and stored in liquid nitrogen. Cells were grown in Dulbecco’s modified Eagle’s medium (DMEM), supplemented with 25 mM HEPES solution, 10% fetal bovine serum, 1% penicillin (5000 µ/mL), 1% l-glutamine (200 mM) (ThermoFisher Scientific, Loughborough, UK) and 1% MEM non-essential amino acids solution (Sigma-Aldrich, Gillingham, UK). Cells were maintained at 37 °C in a humidified incubator containing 5% CO_2_ and 95% air. Cells between passages 30–36 were seeded onto collagen-coated 6-, 12-, 24- or 96-well plates (Bio-Greiner, Stonehouse, UK) at a density of 5 × 10^4^ cells/cm^2^ depending on the experiment and the media was replaced every 2 days. For all experiments, cells were post-confluent and used at 13–15 days post-seeding. In order to ensure low basal iron levels, 24 h prior to the initiation of the experiments, the DMEM medium was replaced with Eagle’s minimum essential medium (MEM), without fetal bovine serum, and supplemented with 10 mmol/L PIPES (piperazine-*N,N’*-bis-(2-ethanesulfonic acid)), 26.1 mM NaHCO_3_, 19.4 mmol/L glucose, 1% antibiotic-antimycotic solution, 11 µmol/L hydrocortisone, 0.87 µmol/L insulin, 0.02 µmol/L sodium selenite (Na_2_SeO_3_), 0.05 µmol/L triiodothyronine and 20 µg/L epidermal growth factor as previously reported [16]. 

LIF was boiled for 10 min in 1 L of Milli-Q (18.2 MΩ) H_2_O at acidic pH (pH 2) for maximal iron release. An acid-washed beaker was used to avoid external iron contamination. Samples of 25 mL were placed in polypropylene tubes, cooled to room temperature, and ascorbic acid (AA) added to obtain a final concentration of 0, 1 and 10 mM, respectively. The pH of the solutions was gradually increased to 7 with 0.1 M NaHCO_3_. The iron released from LIF (LIF-Iron) with or without added AA was determined at this stage prior to further dilution in MEM (LIF-Iron:MEM, 1:1, 1:3, or 1:10, depending on the nature of each experiment). Subsequently, cells were exposed to the different treatments for the indicated times. For iron uptake experiments, Caco-2 cells were subjected to the LIF-iron treatments and to a set of controls including blanks with/without AA and a positive control (0.05 mM FeSO_4_ plus 0.5 mM AA, (FeSO_4_)). When simulated digestion was performed, different methods were used (see next section).

### 2.3. In Vitro Simulated Gastrointestinal Digestion

The simulated gastrointestinal digestion was performed as described by Glahn et al. [16] with minor modifications to adjust for the addition of iron from LIF. A pH 2 saline solution (140 mmol/L NaCl, 5 mmol/L KCl) was used to initiate the simulated digestions. For all the experiments, the saline solution without any added iron was used as a blank digestion control to ensure no iron contamination in the in vitro digestion/cellular system. Additionally, 1 g of freeze-dried peas (containing 51 µg Fe/g dry weight, analysed by ICP-OES as previously described [13]) was added to the saline solution as a reference digestion of the pea matrix sample. To ensure that all of the iron released from the peas during digestion remained in solution when the pH was increased to duodenal levels, ascorbic acid (AA) was added at the gastric step of digestion at a final concentration of 0.5 mM (molar ratio of 1:10, Fe:AA). LIF was boiled for 10 min in 1 L of the pH 2 saline solution and samples of 10 mL were used for digestions (see below). To evaluate the effect of the pea matrix on LIF-iron bioavailability, 10 mL of LIF-iron samples was added to 1 g of pea sample. The impact of dietary iron inhibitors (as found in a meal) on LIF-iron bioavailability was examined by adding tannic acid (TA) or phytic acid (PA) at 0.05 and 0.5 mM, as indicated. 

To simulate gastric conditions, pepsin (0.04 g/mL) was added and the samples were incubated for 60 min on a rolling table at 37 °C. After 60 min, the pH of the samples was gradually adjusted to pH 5.5 with 0.1 M NaHCO_3_, and bile (0.007 g/mL) and pancreatin (0.001 g/mL) enzymes were added. The samples were further readjusted to pH 7, and incubated for 30 min on a rolling table at 37 °C to mimic intestinal conditions. At the end of the simulated gastrointestinal digestion, 1.5 mL of the digestate was placed on top of an upper chamber consisting of a Transwell insert fitted with a 15 KDa molecular weight cut-off dialysis membrane (Spectra/Por 7 dialysis tubing, Spectrum laboratories, Europe) suspended over Caco-2 cell monolayers grown in collagen-coated 6-well plates. The digestates were incubated with the cells for 2 h at 37 °C in a humidified incubator containing 5% CO_2_ and 95% air. Inserts were removed, an additional 1 mL of supplemented MEM was added, and cells were incubated for a further 22 h prior to harvesting for ferritin analysis.

### 2.4. Analysis of Soluble and Total Iron Released from LIF

The total iron content of freshly prepared LIF-iron solution was measured using Ferene-S (3-(2-Pyridyl)-5,6-bis(5-sulfo-2-furyl)-1,2,4-triazine disodium salt hydrate), which binds ferrous iron, forming a deep blue complex. Freshly prepared solutions of LIF-iron were used to determine total iron. The soluble iron content was determined by centrifuging 1 mL aliquots of LIF-iron solution at 10,000 g for 5 min, and supernatants were collected for iron analysis. Samples (100 μL) were digested in 100 μL 1% HCl for 10 min in a shaker water bath at 80 °C. After 10 min, samples were briefly cooled on ice and the following reagents were added sequentially and vortexed after each addition: 500 μL 7.5% ammonium acetate, 100 μL AA, 100 μL 2.5% sodium dodecylsulphate (SDS), and 100 μL 1.5% ferene. Samples were centrifuged at 13,400× *g* for 5 min and the absorbance of the supernatant was measured at 593 nm against an iron standard curve (0–20 nmol Fe as ammonium iron (II) sulfate).

### 2.5. Determination of Ferritin Formation

Ferritin formation was measured 24 h after treatment. Cells were rinsed with Milli-Q (18.2 MΩ) H_2_O and subsequently lysed by scraping in 100 µL (12-well plates) or 200 µL (6-well plates) of CelLytic M (Sigma-Aldrich, Gillingham, UK). Cell lysates were kept on ice for 15 min and stored at −80 °C. For analysis, samples were thawed and centrifuged at 14,000× *g* for 15 min. Cellular debris was discarded and the supernatant containing the proteins was analysed for ferritin using the Spectro Ferritin ELISA assay (Ramco Laboratories Inc., Stafford, TX, USA). The ferritin concentration in the samples was determined using a microplate reader at an excitation wavelength of 500 nm according to the manufacturer’s protocol. Ferritin concentrations were normalized to total cell protein using the Pierce Protein BCA protein assay (ThermoFisher Scientific, Loughborough, UK). 

### 2.6. Determination of Cellular Viability 

Cell viability was determined using the CellTiter 96^®^ Aqueous One Solution colorimetric assay (Promega, Southampton, UK) according to the manufacturer’s protocol. This method is based on the measurement of the colored product of MTS tetrazolium, which is bio-reduced by cells into formazan. NADPH or NADH produced by dehydrogenase enzymes facilitates the bio-reduction in metabolically active cells. Briefly, Caco-2 cells seeded in 96-well plates and grown for 14 days, were treated with the LIF treatments for 24 h. A cell lysis solution, Triton-X (10%), was used as a positive control to produce physical disruption of cell membranes and subsequent cell death. After 24 h, treatments were removed, replenished with fresh MEM containing 20% MTS solution, and cells were incubated for 15 min, prior to reading the absorbance of each well using a microplate reader at 490 nm. 

### 2.7. Determination of the Reactive Oxygen Species (ROS) Generation

Cellular ROS generation was determined using the dichlorofluorescin-diacetate (DCFH) assay as previously described [17] with minor modifications. Caco-2 cells were seeded in collagen-coated 24-well plates and grown for 12 days. On the day prior to LIF treatments, the media was replaced with MEM. On the day of the experiment, 10 µM of DCFH was added to each well for 30 min at 37 °C. Cells were washed with PBS and treated with LIF-iron (with or without AA) diluted in MEM (LIF-iron:MEM, 1:10) or FeSO_4_ in equimolar concentrations (100 µM Fe). After being oxidized by intracellular oxidants, DCFH converts to dichlorofluorescein and becomes fluorescent. ROS generation was measured over time (up to 2 h) using a fluorescent microplate reader with an excitation of 485 nm and an emission of 530 nm. 

### 2.8. Analysis of Iron Content in the Cellular Lysates Samples

The content of iron in the cellular lysates were determined using an Inductively Coupled Plasma Optical Emission Spectroscopy (Varian Vista Pro CCD Axial simultaneous ICP-OES) equipped with a glass expansion Seaspray concentric nebulizer (2 mL/min sample flow rate), a 50 mL glass cyclonic spray chamber and an Axial torch with a 2.3 mm i.d. quartz injector. Sample solutions were introduced using a SDS5 Autosampler. White/white and Blue/Blue PVC acct pump tubing was used. Running conditions are described in Supplementary Table S1. The cellular lysates were 4-fold diluted HNO_3_ (10%) to a final acid concentration of 7.5%. Then, samples were centrifuged 14,500× *g* for 10 min and the supernatants were used for the analysis. Blank controls and internal quality controls were prepared alongside the cell lysates and analysed with the samples. A series of external calibration standards containing iron were prepared from commercial standard stock solutions (Centi Prep), with final concentrations ranging from 0 to 1000 ppb in a diluent with a final concentration of 7.5% HNO_3_. The iron concentrations were calculated against the linear regression obtained from the calibration standards at wavelength of 259.9 nm.

### 2.9. Statistical Analysis

Data are presented as mean values with the standard errors of the means (SEM). Homogeneity of variances was evaluated by the test of Levene. For multiple comparisons, one-way ANOVA followed by a Bonferroni test was used when variances were homogeneous or by Tamhane test when variances were non-homogeneous. Statistical significance was set at *p* ≤ 0.05. The statistical analysis was performed using the SPSS package (version 23; SPSS Inc., Chicago, IL, USA).

## 3. Results

### 3.1. Effect of pH and AA on the Quantity of Iron Released from LIF

**Figure 2:**
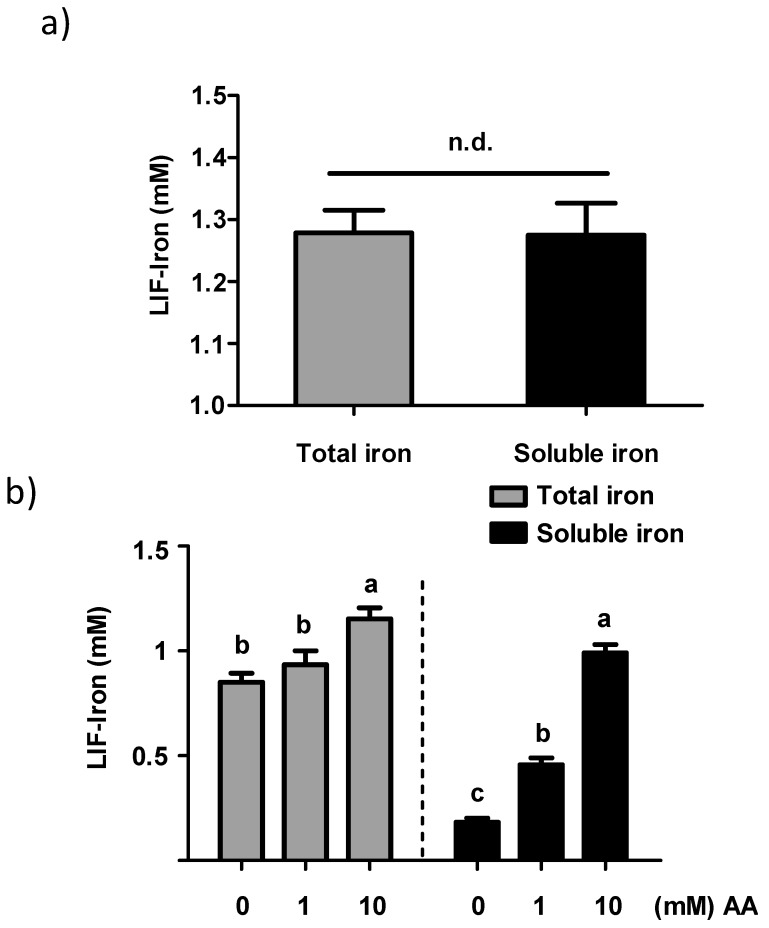
Concentration of total and soluble iron from the iron ingot (Lucky Iron Fish™ (LIF)) at (**a**) pH 2 and (**b**) pH 7 with or without AA. Data represent means ± SEM (*n =* 4). Means without a common letter differ (*p <* 0.05). n.d. means not statistically different.

To evaluate the reproducibility of iron released from LIF, four independent iron extractions were performed in 1 L of water at pH 2. As shown in Figure 2a, similar iron concentrations with a mean of 1.2 mM were obtained at pH 2. However, when the LIF solution was increased to pH 7, a 25% reduction was observed in the total iron concentration. The addition of AA at 1 and 10 mM produced a concentration-dependent increase in the soluble iron in water treated with LIF, from 2.5 to 5.4 fold respectively (Figure 2b). In addition, when the pH of the water was increased from 2 to 7, the addition of 10 mM of AA prevented the precipitation of iron from LIF. 

### 3.2. Effect of Iron Released from LIF on Cell Viability

**Figure 3:**
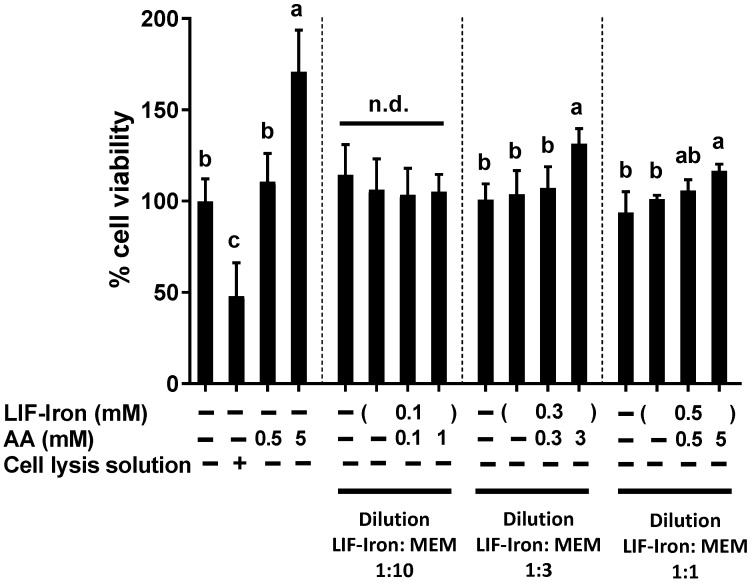
Effect of iron ingot (Lucky Iron Fish™ (LIF)) on cellular viability. Caco-2 cells were treated with the iron released from LIF (LIF-iron) plus the final indicated concentration of ascorbic acid (AA) diluted in MEM (LIF-iron:MEM, 1:10, 1:3 and 1:1) for 24 h. Data represent means ± SEM (*n =* 8). Different letters indicate statistically significant differences (*p <* 0.05). n.d. means not statistically different.

Next, we investigated whether the soluble iron released from LIF in water resulted in changes to the viability of the Caco-2 cell monolayer. As shown in Figure 3, the addition of iron from LIF with AA at different molar ratios Fe:AA, (1:0, 1:1 and 1:10) did not induce changes in cell viability when the LIF treatments were 10-fold diluted in MEM (LIF:MEM, 1:10). However, a modest increase in cell proliferation (30% and 16%) was observed when cells were treated at higher concentrations of LIF-iron with AA (molar ratio, Fe:AA (1:10)) using less diluted treatments (dilution LIF:MEM, 1:3 and 1:1). This increase in cell proliferation was even more pronounced in cells treated only with AA at the highest concentration (5 mM), highlighting the proliferative effects of AA on differentiated Caco-2 cells. Therefore, subsequent experiments using AA were performed at concentrations ≤1 mM to avoid any effects on cellular proliferation. 

### 3.3. Effect of AA on Cellular Iron Uptake from LIF

**Figure 4:**
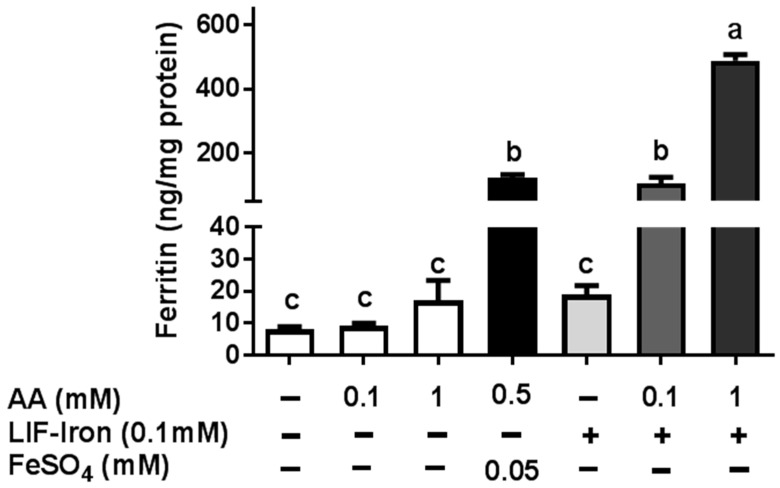
Cellular ferritin response, as a surrogate of the iron uptake, from the iron ingot (Lucky Iron Fish™ (LIF)) with or without ascorbic acid (AA). Caco-2 cells were exposed for 24 h to the LIF-iron (0.1 mM Fe) with the indicated concentration of AA. Data represent means ± SEM (*n =* 6–8). Different letters indicate statistically significant differences (*p <* 0.05).

In order to investigate whether the increase in soluble iron associated with AA was bioavailable to intestinal cells, the cellular ferritin response, a surrogate marker of iron uptake, was measured in Caco-2 cells (Figure 4). No significant difference was found between blank controls with/without AA at 0, 0.1 and 1 mM, with 8.1, 9.1 and 17.1 ng/mg of protein respectively, whereas a high ferritin response (122 ng/mg of protein) was observed for FeSO_4_. LIF treatment without AA did not result in a significant increase in the ferritin response (18.9 ng/mg of protein). However, the addition of AA in 0.1 and 1 mM amounts to LIF significantly increased the ferritin response by 100 and 480 ng/mg of protein respectively. In addition, the analysis of the iron content of the Caco-2 cell lysates by ICP-OES confirmed the effect of AA on the cellular iron uptake from LIF (Supplementary Figure S1).

### 3.4. Effect of Including Food Matrix Dietary Factors in a Simulated Gastrointestinal Digestion on Cellular Iron Uptake from LIF

**Figure 5:**
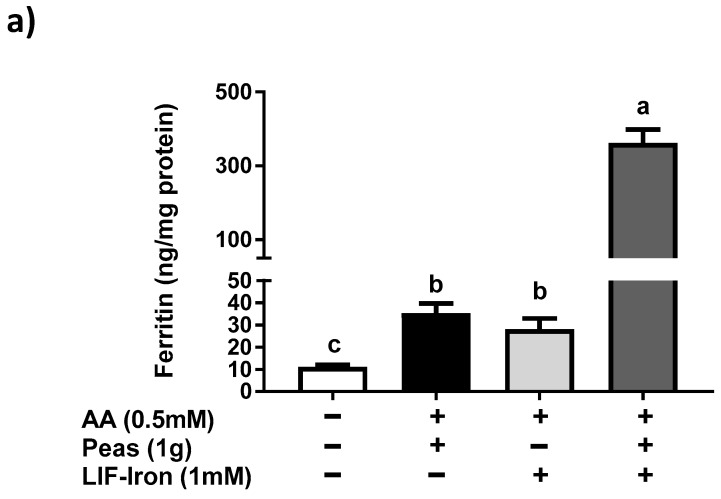

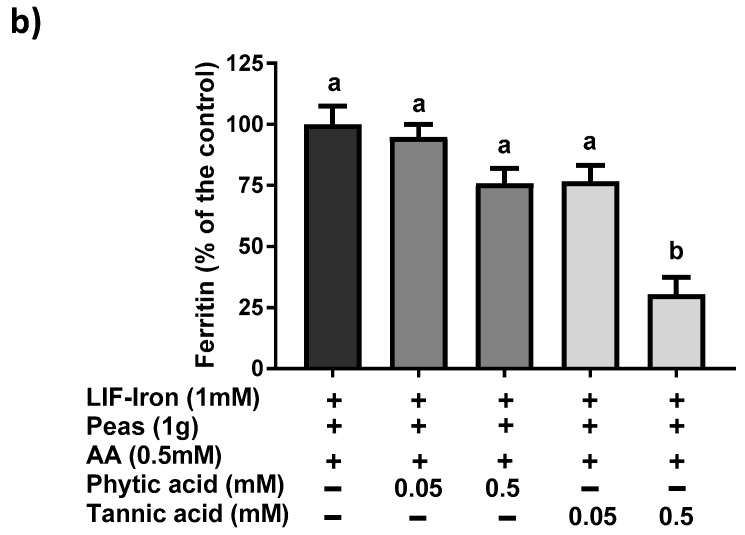
Iron uptake in Caco-2 cells exposed to simulated gastrointestinal digestates of peas and different dietary factors combined with the (Lucky Iron Fish™ (LIF))-iron. Cellular ferritin response exposed for 24 hour incubation with the in vitro gastrointestinal digestion containing LIF-iron plus ascorbic acid (0.5 mM) (**a**) with or without 1g of pea; and (**b**) with pea plus added tannic acid or phytic acid at the indicated concentrations. Data represent means ± SEM (*n =* 6–8). Means without a common letter differs (*p <* 0.05).

LIF is designed for home fortification of cooked foods. We thus assessed the impact of a pea food matrix (a common staple food) on the iron uptake from LIF in Caco-2 cells after an in vitro digestion. Cells were exposed to digestates of LIF, peas or the combination of LIF plus peas as presented in Figure 5a. We found a significantly increased ferritin response with LIF and peas compared to the blank control (35 and 28 vs. 11 ng/mg of protein, respectively). Surprisingly, the combination of LIF plus peas produced an increase in ferritin response by about 10-fold compared to the treatment in isolation (Figure 5a and Supplementary Figure S2). 

In order to simulate the effect of a mixed-diet containing iron chelators, we added phytic and tannic acid, two well-known dietary inhibitors of iron absorption, to the in vitro digestion containing the combination of LIF plus peas (Figure 5b). We found that tannic acid at 0.5 mM reduced the ferritin response from LIF plus peas by 75%, but no significant changes were observed at a lower concentration of tannic acid or at any concentration of phytic acid. 

### 3.5. Effect of Iron Released from LIF on ROS Generation

**Figure 6:**
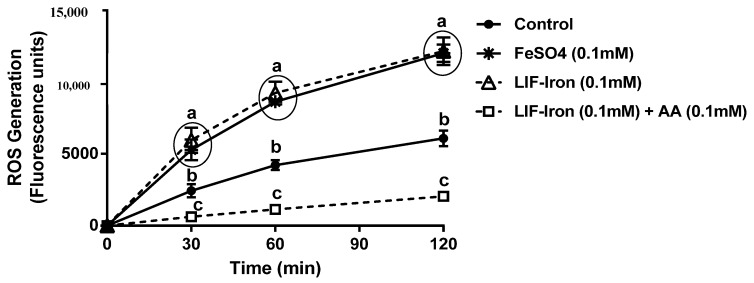
Effect of the iron ingot (Lucky Iron Fish™ (LIF)) on reactive oxygen species (ROS) generation. Cells were exposed to FeSO_4_ (0.1 mM) or a similar LIF-iron concentration with or without AA. The intracellular ROS production was evaluated at 0, 30, 60 and 120 min. Data represent means ± SEM (*n =* 8). Different letters indicate statistically significant differences at each time point (*p <* 0.05).

Finally, we explored the possibility that the iron released from LIF could generate oxidative stress similar to FeSO_4_, a widely used iron supplement. We observed that the addition of iron from LIF induced a 2-fold increase in ROS generation after 30 min, which was similar to FeSO_4_ (added in a similar iron concentration (0.1 mM)). These levels were sustained for at least 2 h. However, the addition of AA, with its potent antioxidant behaviour, at a Fe:AA molar ratio of 1:1, significantly reduced oxidative stress caused by the iron released from LIF (Figure 6).

## 4. Discussion

In this study, we provide the first direct evidence of the potential bioavailability of iron from the LIF home fortificant, using a widely used in vitro digestion/Caco-2 cell model for assessing iron uptake at the intestinal level. Furthermore, we explored the effect of dietary factors and an example of a food matrix on iron uptake from LIF. In particular, we found (1) a dose-response with AA in relation to iron solubility and iron uptake in Caco-2 cells from LIF; (2) a high ferritin response from LIF in the presence of peas when they were subjected to simulated digestion; and (3) a reduction in iron availability from LIF when tannic acid was added to the digestion. Finally, we demonstrated that LIF induced an overproduction of ROS, similar to FeSO_4_, which was counteracted by AA without causing cellular cytotoxicity at the concentrations used in our cellular model. 

Previous studies investigating the total iron released by LIF in water at different pH values reported that the amount of iron was higher at lower pH [9,10]. Armstrong et al. [10] reported that LIF released similar amounts of iron (about 80 µg/mL Fe) in acidic conditions (pH 3.5) towards more neutral pH conditions. However, at pH 7, the total iron released significantly decreased to 30 µg/mL Fe. The amount of iron released in our experiments was in agreement with this study, with 1.2 mM (67 µg/mL) and 0.85 mM (47 µg/mL) of iron at pH 2 and pH 7, respectively. There is also evidence that water weakly acidified (pH 3.2–4.5) with lemon juice or other foods, can have a differential effect on the quantity of iron released from LIF [9]. However, specific dietary factors or the extent to which these factors impact LIF iron solubility have not been investigated. We studied the influence of AA, an enhancer of iron absorption, on the soluble and total iron released from LIF in water. We observed that AA facilitated iron solubility in water with increasing pH (pH 2 to pH 7) in a concentration-dependent manner, providing further evidence for the potential efficacy of AA on maintaining LIF-iron in a form that can be absorbed in the small intestine. 

Three RCTs [7,8,11] have been carried out in Cambodia investigating the effect of using the LIF ingot in food and drinking water. Results from these trials were conflicting. In the first trial, LIF significantly improved the hemoglobin levels of women after 3 months, but these levels reverted back to baseline after 6 months [8]. In the follow-up trial, both hemoglobin and serum ferritin were measured at 3, 6, 9 and 12 months. LIF increased hemoglobin levels after 9 months (118 vs. 123 g/L) and both hemoglobin (120 vs. 130 g/L) and serum ferritin (66 vs. 102 ng/mL) levels after 12 months [7]. The authors suggested that the lack of efficacy of LIF at 6 months in the first RCT might be due to seasonal variations in the water parameters that could reduce iron bioavailability. A third RCT reported no changes in hemoglobin after 6 or 12 months of using LIF [11]. However, in this trial the prevalence of structural hemoglobin variants was about 70%, and only 9% of participants had serum ferritin concentrations indicative of iron deficiency, which suggests that this population was not ideal for evaluating the efficacy of LIF. 

As far as we are aware, no studies have examined whether the iron released from LIF itself is bioavailable in the small intestine after exposure to gastrointestinal digestion. This requires the use of in vitro models to assess the impact of different dietary factors on iron uptake from LIF. Here, we have demonstrated that while LIF released a high amount of iron in acidic conditions, it is poorly bioavailable in Caco-2 cells in the absence of iron enhancers at neutral pH conditions found in the intestine. Nevertheless, the addition of AA increased the amount of soluble iron and iron uptake in Caco-2 cells. Yun et al. [15] examined the effect of AA on iron bioavailability, comparing the Caco-2 cell response with previously published human absorption data. In their study, AA ranging from 25 to 500 mg added to semisynthetic meals, increased iron absorption in Caco-2 cells and was predictive of its effect in human trials. Hence, we suggest that the addition of AA to LIF during food preparation can have a major role in improving iron absorption in vivo, especially in iron-deficient populations.

In order to determine the impact of the food matrix on iron bioavailability from LIF, we undertook a simulated gastrointestinal digestion with peas (a staple food) plus LIF-iron. We observed that this combination produced a much greater (10-fold) ferritin response in cells compared to pea and LIF-iron individually. According to its nutritional composition [18], peas contain about 0.22 mg/g of AA, so it is highly unlikely that the quantity of endogenous AA provided from the peas could account for more than 0.5 mM in our simulated digestion. Therefore, we suggest that other dietary factors from the pea matrix contributed to the increase in iron uptake of the digestate with LIF-iron and peas. For example, peas contain a high amount of sucrose (66 mg/g fresh weight) [18]. There is evidence for an enhancing effect from sugars, and in particular fructose, on iron uptake in Caco-2 cells [19]. Thus, it is possible that some sucrose could be hydrolysed into glucose and fructose by the sucrase activity of differentiated Caco-2 monolayers [20] thereby enhancing iron absorption. However, we cannot rule out other dietary factors from the pea matrix, which can result in promoting iron absorption. Considering the high levels of total iron from LIF in the digestates (approximately 1 mM), even a small amount of enhancer would result in a considerable quantity of iron being taken into the cells. Further research is warranted to elucidate the reasons for the unexpected positive interaction between peas and LIF. The addition of exogenous (0.5 mM) tannic acid but not phytic acid to the simulated digestion mixture reduced iron uptake by 75%, which is in agreement with studies which showed that tannic acid is a much more potent inhibitor of non-heme iron uptake than phytic acid in Caco-2 cells [21]. All of these data suggest that the iron bioavailability from LIF can be modulated differently depending on the dietary factors present in the food matrix during cooking and digestion.

The manufactured LIF ingot contains a mixture of predominately ferrous iron, trace amounts of ferric iron, and iron complexed to other minerals [10]. Forms of ferrous iron are widely used as oral supplements due to their relative high bioavailability. However, they are also associated with gastrointestinal side effects, which result in non-adherence to the treatment [22,23]. Oxidative stress generated by ferrous iron salts has been proposed as one of the main reasons for GI intolerance [22,23]. Cellular death was not evident with LIF when cells were exposed to our iron uptake treatments with or without AA. However, we observed that the addition of LIF-iron generated an overproduction of ROS to the same extent as FeSO_4_, which indicates that they have similar iron chemistry. In contrast, the addition of AA (in a molar ratio Fe:AA 1:1) ameliorated the increase in ROS generation. There is evidence suggesting that intracellular ROS generation induced by iron could be modulating divalent metal transporter-1 (DMT-1) internalization as a redox sensor to control iron uptake [24]. Esparza et al. [24] demonstrated that DMT-1 internalization induced by Fe^2+^ was prevented by pre-incubation with the antioxidant *N*-acetyl-l-cysteine (NAC), suggesting that iron-induced ROS was counteracted by NAC. This is in agreement with our results. Thus, the most plausible explanation is that free iron transported inside the cells produced an increase in ROS, which in turn internalised DMT-1 and reduced iron uptake. However, by complexing/binding ferrous iron [25] or neutralising free radicals [23], AA is likely to have prevented the intracellular environment from further oxidation, resulting in the increase in iron uptake. Therefore, all the above suggests that AA could enhance iron uptake not only through an increase in soluble iron but also through intracellular redox mechanisms that are DMT-1 dependent. 

Despite the fact that LIF could ameliorate iron deficiency in the short term, especially if our findings are taken into account when providing instructions for the use of LIF, further studies on the long-term effect of this iron fortificant must be performed to assess possible adverse consequences. For example, the daily use of micronutrient powders for four months as an in-home fortification strategy has been associated with changes in the gut microbiome profile of weaning infants and an increased abundance of enteropathogens bacteria, which in turn was associated with inflammation [26]. Likewise, the use of home-fortification strategies should be very tightly controlled in anemic populations where genetic hemoglobin disorders (i.e., mild thalassemia) or inflammation, rather than dietary iron deficiency, are the main causes of anemia, as in such cases it could lead to iron overload [27,28,29].

In conclusion, this study demonstrates that dietary factors can modulate the solubility and bioavailability of LIF-iron forms. The addition of AA resulted in greater iron solubility, was associated with lower ROS production, and enhanced iron uptake in Caco-2 cells. The wider use of AA and the selection of foods with recognised iron enhancing properties in the guidelines for LIF might help to make this strategy more effective for reducing iron deficiency. 

## Supplementary Materials

The following are available online at www.mdpi.com/2072-6643/9/9/1005/s1, Figure S1: Iron content in cellular lysates of Caco-2 cells after treatment with the iron ingot (Lucky Iron Fish^TM^ (LIF)) with or without ascorbic acid (AA), Figure S2: Iron content in cellular lysates of Caco-2 cells exposed to simulated gastrointestinal digestates of peas combined with the (Lucky Iron Fish^TM^ (LIF))-iron, Table S1: Running conditions used for ICP-OES.
