# Supplementary material for: Dietary Factors Modulate Iron Uptake in Caco-2 Cells from an Iron Ingot Used as a Home Fortificant to Prevent Iron Deficiency
Source: Nutrients. 2017 Sep 12;9(9):1005. doi: 10.3390/nu9091005 (PMC5622765; doi:10.3390/nu9091005)
Supplement: Supplementary file 1 [file nutrients-09-01005-s001.zip › nutrients-220512-supplementary.pdf]

Table S1. Running conditions used for ICP-OES

Analytical Conditions	Fe
RF power (W)	1200
Plasma gas (L min^{-1})	15
Auxiliary gas (L min^{-1})	1.50
Speed pump (rpm^{1})	10
Nebulizer gas flow rate (L/min^{-1})	1.05
Instrument stabilization (sec)	10
Replicate read time (sec)	5

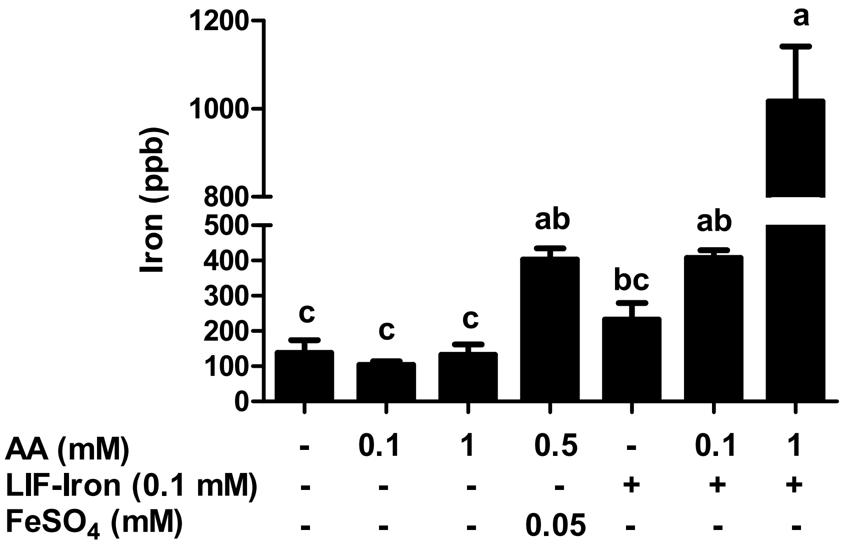

Supplementary Figure S1. Iron content in cellular lysates of Caco-2 cells after treatment with the iron ingot (Lucky Iron Fish™ (LIF)) with or without ascorbic acid (AA). Caco-2 cells were exposed for 24 hours to the LIF-iron (0.1 mM Fe) with the indicated concentration of AA. Data represent means ± SEM (n = 4-6). Different letters indicate statistically significant differences ($p < 0.05$).

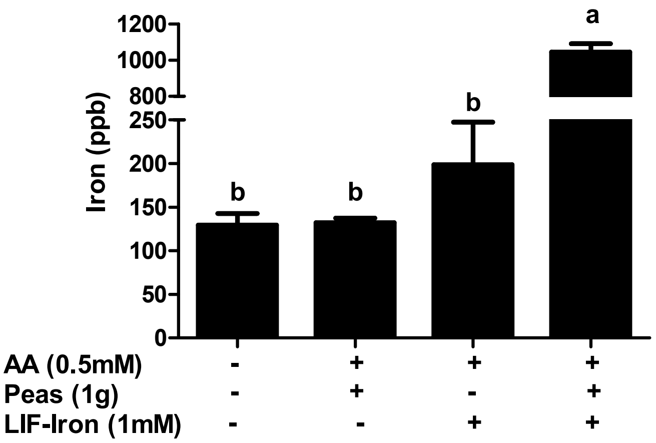

Supplementary Figure S2. Iron content in cellular lysates of Caco-2 cells exposed to simulated gastrointestinal digestates of peas combined with the (Lucky Iron Fish™ (LIF))-iron. Caco-2 cells were exposed for 24 hour incubation with the in vitro gastrointestinal digestion containing LIF-iron plus ascorbic acid (0.5 mM) with or without 1g of pea. Data represent means ± SEM (n = 3-4). Means without a common letter differ ($p < 0.05$).
